# Anti-Neutrophilic Cytoplasmic Antibody (ANCA) Vasculitis Presented as Pulmonary Hemorrhage in a Positive COVID-19 Patient: A Case Report

**DOI:** 10.7759/cureus.9643

**Published:** 2020-08-10

**Authors:** Albadr Hussein, Khaled AL Khalil, Yasser M Bawazir

**Affiliations:** 1 Medicine/Rheumatology, King Fahad General Hospital, Madina, SAU; 2 Internal Medicine/Rheumatology, King Abdulaziz University, Jeddah, SAU

**Keywords:** anca, granulomatosis with polyangiitis, alveolar hemorrhage, covid-19

## Abstract

Granulomatosis with polyangiitis is a small vessel vasculitis with a wide spectrum of presentation, ranging from limited disease to life-threatening situation such as alveolar hemorrhage. Immunosuppression is the corner stone of the treatment and if left untreated, the death toll increases dramatically. We presented a case of granulomatosis with polyangiitis, presented with alveolar hemorrhage associated with COVID-19 infection. The patient admitted to the intensive care unit, received pulse steroids, plasmapheresis and intravenous immunoglobulin. She was not given further immunosuppression because of the coexisting COVID-19. Up to our knowledge, this is the first reported case of alveolar hemorrhage secondary to granulomatosis with polyangiitis coexisting with COVID-19 infection.

## Introduction

Anti-neutrophilic cytoplasmic antibodies (ANCA) are autoantibodies directed against the cytoplasmic granules of the monocytes and neutrophils. It can be detected by using the immunofluorescence staining. If the stain reacts with the cytoplasm it will be cytoplasmic antineutrophilic cytoplasmic antibody (C-ANCA) and if the stain reacts with perinuclear granules it will be known as perinuclear antineutrophilic cytoplasmic antibody (P-ANCA). Studies showed by enzyme-linked immunosorbent assay (ELISA), the target antigen for the P-ANCA is myeloperoxidase (MPO) while for the C-ANCA is proteinase 3 (PR 3) [1].

Indeed, ANCA antibodies are useful for the diagnosis of small vessel vasculitis including granulomatosis with polyangiitis (GPA), microscopic polyangiitis (MPA) and eosinophilic granulomatosis with polyangiitis (EGPA). Moreover, positive PR 3 presents in almost 70-80% of cases with GPA [2].

Granulomatosis with polyangiitis is uncommon form of vasculitis characterized by severe inflammatory reaction and it affects the upper airways, lung and kidneys. It is common among Caucasians with prevalence of five cases per 100,000 population in Europe. Males and females are equally affected [3]. Patients with lung involvement usually present with cough, shortness of breath, pleuritic chest pain and hemoptysis. There is a significant mortality risk in patients presented with alveolar hemorrhage duo to respiratory failure. The diagnosis of GPA is based on the clinical presentation, ANCA serology and histopathology of the involved organ [4].

Several organisms are associated with development of GPA with positive C-ANCA - PR3, the most commonly known organism is Staphylococcus aureus (S. aureus). Sixty to seventy percent of the patients are carrier of this organism in their nasal canals. Apart from S. aureus, other organisms are: Streptococcus pneumoniae, Haemophilus influenzae, Klebsiella pneumoniae, hepatitis C virus (HCV), Epstein-Barr virus (EBV) and Helicobacter pylori [5, 6].

The treatment of GPA depends on the severity of the presentation based on immunosuppression. In life-threatening cases, several regimens are proposed including combination of high-dose corticosteroid with cyclophosphamide or rituximab [4]. Plasma exchange is used as supplementing treatment in GPA but based on the recent PEXIVAS trial it is not helpful in reducing the morbidity or mortality [7].

GPA mortality rate ranges between 12.5 and 25.7%; if left untreated it reaches up to 82% in the first year [8].

To our knowledge, there is no case of ANCA vasculitis associated with positive corona virus disease 2019 (COVID-19). We report a case of ANCA positive GPA associated with a positive COVID-19.

## Case presentation

A 37-year-old Saudi female, who is not known to have any medical illness, presented to the emergency department with chest pain, cough, shortness of breath, hemoptysis and generalized joint pain. Her symptoms progressed over the last four days. There was no history of fever, skin rashes, sicca symptoms, no uveitis, no oral or genital ulcers. She denied any history of drug abuse and alcohol intake. She is not a smoker. There was no history of previous miscarriages and thrombovenous embolism.

The patient was admitted to isolation section in the emergency department (ER). Her Glasgow coma scale (GCS) was 15, temperature 37 C, blood pressure 110/65, respiratory rate 20, heart rate 120 bpm. Oxygen saturation on room air was 90% improved to 96% on high flow oxygen. Heart sounds were normal, no murmurs, jugular venous pressure (JVP) not raised, bilateral lower limb pedal pitting edema, bilateral equal breathing sound with audible crackles in both lungs and no tenderness or guarding in the abdomen. Musculoskeletal exam showed active arthritis in the 2nd, 3rd and 4th proximal interphalangeal joints of the right hand.

**Figure 1:**
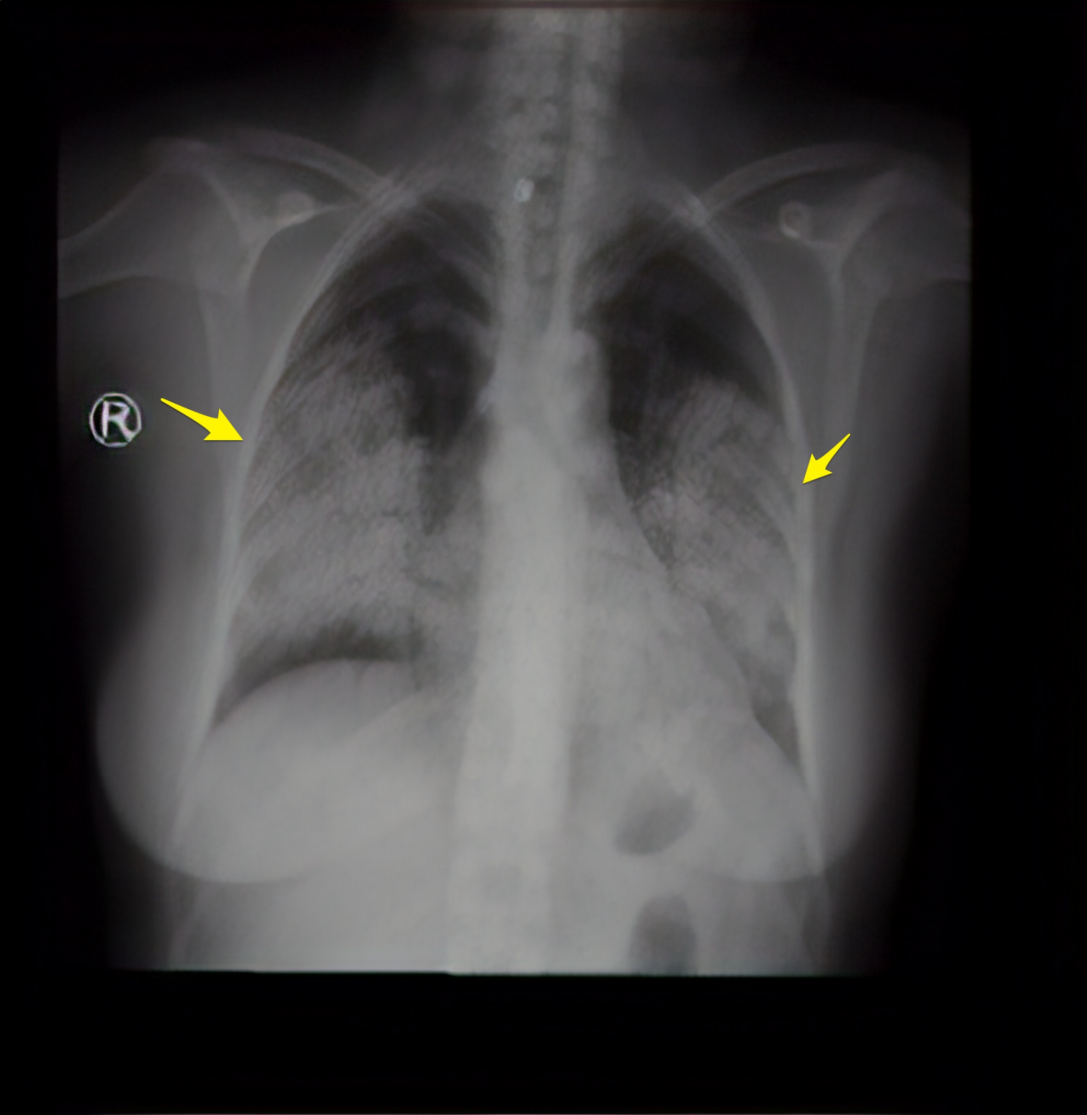
Chest X-ray (PA view) Posteroanterior (PA) view: diffuse bilateral middle and lower zone heterogeneous opacity (Yellow arrows).

**Figure 2:**
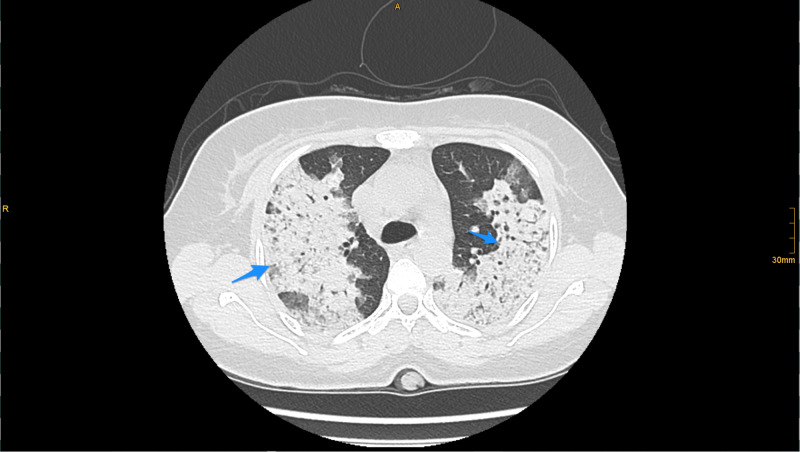
CT chest (lung window) CT chest (lung window) demonstrated a widespread patchy consolidation with central and peripheral distribution, permeated by ground glass areas and crazy paving pattern. These findings are suggestive of diffuse alveolar hemorrhage (blue arrows).

**Figure 3:**
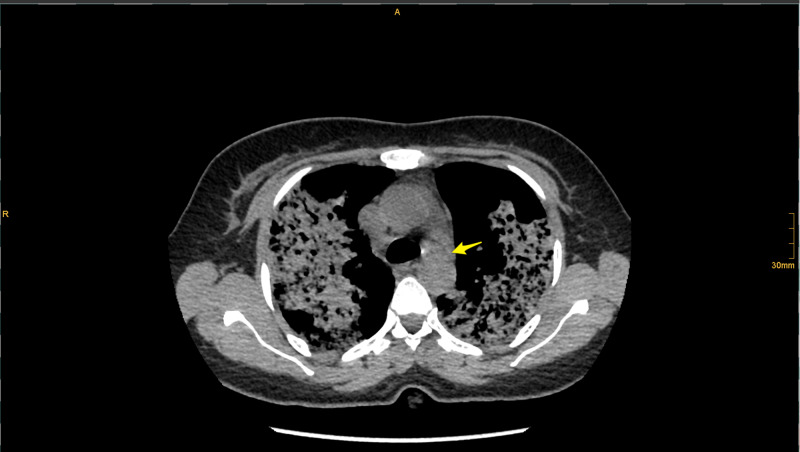
CT chest (Mediastinal window) The evaluation is limited but there are a few paratracheal lymph nodes (yellow arrow).

Initial hematological examination revealed white blood cells (WBC) 13,000 cells/µL, hemoglobin 5.2 g/dL, platelets 414,000 u/L, normal coagulation profile, glucose 9.1 mmol, creatinine 284 µmmol/L, blood urea nitrogen (BUN) 10.9 mmol/L, sodium (Na) 134 mmol/L, potassium (K) 3.8 mmol/L, chloride (Cl) 96 mmol/L, lactic acid 1.8 mmol, albumin 35 g/L; liver function tests were normal, cardiac enzymes and electrocardiogram were normal, C-reactive protein (CRP) 252 mg/L; urine analysis and microscopy revealed no red blood cells casts. Chest X-ray revealed bilateral pulmonary infiltration (Figure 1). COVID-19 polymerase chain reaction (PCR) test was negative. CT brain showed no acute brain insults. Chest CT showed a widespread lung patchy consolidation with a central and peripheral distribution, permeated by ground glass areas and crazy paving pattern. The apices lower zones were relatively spared. Findings were likely suggestive of diffuse alveolar hemorrhage (Figures 2-3).

Patient's blood, urine and wound cultures were taken. Toxicology screen was negative. The patient was stabilized, received proper hydration, 2 units packed red blood cells and broad-spectrum antibiotics initiated. Her serology showed that C-ANCA (PR3) 94.79 AU/mL (strong positive), P-ANCA 7.88 AU/mL (negative), antinuclear antibody (ANA) mild positive 1/80. Her complement C3 and C4 levels were within normal, the rest of rheumatological workup was unremarkable. At this point intravenous pulse methyl prednisone was initiated for three days.

The patient was shifted to the intensive care unit (ICU), as she had ongoing hemoptysis and her oxygen saturation dropped to 84% on BiPAP with FiO2 75%. After she completed the pulse steroids, prednisone 60 mg daily was initiated; linezolid was added to the antibiotic regimen. Twenty-four hours later, her GCS dropped to 9/15; she was intubated, and she was initiated on six sessions of plasmapheresis.

After she completed the plasmapheresis, the second COVID-19 swab test was positive. At this point, ritonavir/lopinavir was added and antibiotics was changed to imipenem based on infectious disease recommendations. Prednisone was reduced to 30 mg and intravenous immunoglobulin was initiated for a total of five doses.

To confirm the diagnosis of COVID-19 infection, a third sample was taken and it came positive. She was having continuous fever; antifungal treatment was added. Her hemoptysis decreased significantly after the intravenous immunoglobulin (IVIG). After 20 days of ICU admission, she had increased oxygen demands and required more vasopressors, and eventually passed away.

## Discussion

We reported this case in which the initial presentation was pulmonary hemorrhage with positive C-ANCA, suggestive of granulomatosis with polyangiitis vasculitis. The patient had two positive COVID-19 PCR tests. Her presentation was suggestive of severe disease with pulmonary hemorrhage, acute kidney injury. Her treatment was difficult as she needed aggressive immunosuppression which could lead to death by COVID-19 infection.

COVID-19 is a member of the large family of coronavirus, transmitted from human to human by the exposure of the respiratory droplets of symptomatic subject. The spectrum of clinical presentation can be classified as: mild, moderate and severe. Patients with mild symptoms have features of upper respiratory tract infection, moderate case presents with shortness of breath and tachypnea, severe case presents with acute respiratory distress syndrome (ARDS), sepsis and septic shock. The laboratory findings of COVID-19 include high C-reactive protein (CRP), D-dimer, high prothrombin time, high alanine aminotransferase, and lactate dehydrogenase. To confirm the diagnosis of COVID-19, the WHO recommends the polymerase chain reaction (PCR) technique to discover the virus RNA and if the first sample is negative with a high clinical suspicion a second sample is warranted [9].

Subjects with COVID-19 infection, who carry the TMEM173 gene variant, are more likely to have a strong inflammatory reaction through the activation of STING inflammatory pathway. Several reports linked the activation of this pathway with Kawasaki vasculitis activation in COVID-19 infected young subjects [10]. The first reported case of Kawasaki vasculitis associated with positive COVID-19 infection was in a six-month-old infant who was admitted as a classical Kawasaki and later tested positive for COVID-19; she was treated with aspirin and intravenous immunoglobulin (IVIG) and discharged home [11].

Granulomatosis with polyangiitis can affect the upper and the lower respiratory tract and the presence of the COVID-19 pandemic raises many questions about the course of this disease and its treatment. The experience reports suggest that patients with chronic arthritis, receiving biological/synthetic disease modifying antirheumatic drugs (DMARDs) are not at higher risk to have severe COVID-19 compared with normal population. There is no experience of ANCA vasculitis with positive COVID-19. There is only one case of granulomatosis with polyangiitis who developed COVID-19 infection after a maintenance dose of rituximab. The patient recovered after a severe course of COVID-19 [12].

## Conclusions

The clinical significance of our patient with a positive COVID-19 and ANCA positive granulomatosis with polyangiitis is not clear. The COVID-19 PCR was positive twice in our patient which was accurate. In regard to our patient presentation, her disease was life threatening with pulmonary hemorrhage and shock. Although she was treated with IVIG, plasmapheresis and high-dose methylprednisolone, she died. To our knowledge, this is the first case of ANCA positive granulomatosis with polyangiitis and COVID-19. We hope this report can help as a reference for rheumatologist to understand the possible association of COID-19 with different forms of vasculitis.

## References

[1] Bosch X, Guilabert A, Font J (2006). Antineutrophil cytoplasmic antibodies. Lancet.

[2] Csernok E, Lamprecht P, Gross WL (2006). Diagnostic significance of ANCA in vasculitis. Nat Clin Pract Rheumatol.

[3] Gubbels SP, Barkhuizen A, Hwang PH (2003). Head and neck manifestations of Wegener's granulomatosis. Otolaryngol Clin North Am.

[4] Grygiel-Górniak B, Limphaibool N, Perkowska K, Puszczewicz M (2018). Clinical manifestations of granulomatosis with polyangiitis: key considerations and major features. Postgrad Med.

[5] Shimomura M, Morishita H, Meguro T (2016). Chronic active EBV infection with features of granulomatosis with polyangiitis. Pediatr Int.

[6] van Timmeren MM, Heeringa P, Kallenberg CG (2014). Infectious triggers for vasculitis. Curr Opin Rheumatol.

[7] Walsh M, Merkel PA, Peh CA (2020). Plasma exchange and glucocorticoids in severe ANCA-associated vasculitis. N Engl J Med.

[8] Fernández-Ávila DG, Rondón-Carvajal J, Villota-Eraso C, Gutiérrez-Dávila JM, Contreras-Villamizar KM (2020). Demographic and clinical characteristics of patients with ANCA-positive vasculitis in a Colombian University Hospital over a 12-year period: 2005-2017. Rheumatol Int.

[9] Hassan SA, Sheikh FN, Jamal S, Ezeh JK, Akhtar A (2020). Coronavirus (COVID-19): a review of clinical features, diagnosis, and treatment. Cureus.

[10] Berthelot JM, Drouet L, Lioté F (2020). Kawasaki-like diseases and thrombotic coagulopathy in COVID-19: delayed over-activation of the STING pathway?. Emerg Microbes Infect.

[11] Jones VG, Mills M, Suarez D (2020). COVID-19 and Kawasaki disease: novel virus and novel case. Hosp Pediatr.

[12] Guilpain P, Le Bihan C, Foulongne V (2020). Rituximab for granulomatosis with polyangiitis in the pandemic of covid-19: lessons from a case with severe pneumonia. Ann Rheum Dis.

